# Translating new science into the community to promote opportunities for breast and cervical cancer prevention among African American women

**DOI:** 10.1111/hex.12985

**Published:** 2019-12-04

**Authors:** Elisa M. Rodriguez, Lina Jandorf, Julia A. Devonish, Frances G. Saad‐Harfouche, Nikia Clark, Detric Johnson, Anika Stewart, Christy A. Widman, Deborah O. Erwin

**Affiliations:** ^1^ Roswell Park Comprehensive Cancer Center, Cancer Prevention and Population Sciences Buffalo New York; ^2^ Icahn School of Medicine at Mount Sinai New York City New York; ^3^ Witness Project of Long Island Long Island New York

**Keywords:** African American, cancer education, community‐based participatory research, dissemination

## Abstract

**Background:**

New evidence has found breast and cervical cancer risk factors unique to African American women. Thus, there is a significant need to increase their knowledge and understanding of relevant risk factors and the potential protective benefits associated with breast‐feeding and HPV vaccination. The National Witness Project is a robust, evidence‐ and community‐based lay health advisor programme that uses group education, navigation and survivor narratives to increase cancer screening among diverse underserved women.

**Methods:**

A multi‐phase, community‐based participatory research study was conducted across three sites in Buffalo, NY, New York City and Arkansas between October 2016 and January 2017. Pre‐/post‐test surveys were administered during volunteer trainings and community programmes. An evaluation survey was also administered at the Annual Meeting for Education and Networking. Paired sample *t* tests were used to compare pre‐/post‐test survey scores.

**Results:**

Trainee survey results showed the overall mean per cent correct pre‐/post‐test scores were 47.7% (SD: 21.87) and 79.2% (SD: 16.14). Altogether, 31 educational programmes reached 332 community participants. Participants’ breast and cervical cancer knowledge scores were significantly higher after the education programme (84.4%) than before (55.3%) with a mean change score of 29% (*P* ≤ .001).

**Conclusion:**

This paper reveals the underlying complexities to update the educational curriculum content of a multi‐site, community‐based outreach organization. The new curriculum significantly improved African American women's knowledge about breast and cervical cancer by 10%‐36%, clearly demonstrating that this information was new to them. The need for education programming in African American communities to disseminate cancer prevention and risk information remains high.

## 1 INTRODUCTION

Recent data show that the gap in breast cancer incidence between white and African American women is closing.^1^ More specifically, African American women are diagnosed more frequently than previously recognized, yet death rates still remain comparatively much higher.^1^ New scientific research from the African American Breast Cancer Epidemiology and Risk (AMBER) Consortium also suggests that higher parity and lack of breast‐feeding increase African American women's risk of developing ER‐ and triple‐negative breast cancer (TNBC).^2^ The data also demonstrated that this increased risk of TNBC can be mitigated with breast‐feeding.^2^ These findings have the potential to drastically impact the incidence of breast cancer and related outcomes in African American women in the United States as these women are disproportionately affected by ER‐ breast cancer, and compared with white women, they have more children^3^, ^4^ and are less likely to breast‐feed.^5^, ^6^ In addition, following Hispanic women African American women in the United States are also more likely to get cervical cancer^7^ and have a mortality rate twice that seen in white women. Significant racial health disparities persist, despite the decline in cervical cancer rates seen across the United States. African American women are more likely to die of cervical cancer due to a combination of factors including later stage at diagnosis,^8^, ^9^, ^10^, ^11^ less aggressive treatment^11^, ^12^ and less access to care.^10^, ^12^, ^13^ AMBER’s findings necessitate a complete paradigm shift in cancer prevention and control efforts among African American women. There is a significant need to increase breast and cervical health knowledge and understanding among African American women regarding their risk of ever developing breast and cervical cancer, and the potential protective benefits associated with certain health behaviours (ie breast‐feeding and cervical cancer screening).

Significant technological advances and targeted programmes for educating communities have not been fully utilized and are available resources to enhance both the implementation and dissemination of new breast and cervical cancer prevention and risk information. The National Witness Project (NWP) model was developed by Erwin in 1991 in collaboration with African American cancer survivors to reduce breast and cervical cancer disparities among African American women through faith‐ and community‐based educational programmes.^14^, ^15^ NWP is one of the most robust and lasting evidence‐based lay health advisor (LHA) programmes using group education, navigation, empowerment messages and survivor narratives to increase breast and cervical cancer screening among African American women in community settings.^15^, ^16^ The NWP model was developed with a theoretical foundation in health education, learning styles and ethnographic fieldwork.^17^, ^18^ The selected behaviour change theories were especially relevant to address breast and cervical cancer disparities while taking into account the specific cultural and educational needs among rural underserved African American women in Arkansas, where the programme was originally developed. Over the past 28 plus years, the NWP model has been implemented, replicated and disseminated in over 40 sites in 22 states, with over 400 trained volunteers, reaching up to 10 000 women annually.^1^, ^19^ Currently, there are 18 active sites within the NWP network that continue to demonstrate capacity and sustainability for programme implementation. In the 10 years since the NWP was listed on the National Cancer Institute's (NCI) Cancer Control PLANET website as a Research‐Tested Intervention Program, both screening and risk‐reduction guidelines have changed (eg ages and frequency of mammography, de‐implementation of breast self‐examination, availability of human papillomavirus (HPV) vaccine to prevent cervical cancer and risks for TNBC), especially for African American women. These changes have resulted in a significant need to revise the NWP curriculum to disseminate the most recent science and to issue a call‐to‐action for African American women to address their breast and cervical cancer risks, incidence and mortality disparities.

This paper presents the systematic approach used to update the NWP educational programme curriculum and evaluate its effectiveness among participants who attended educational programmes as part of a pilot study. The first objective in this effort was to update and strengthen the NWP educational curriculum to include new scientific content relevant to breast and cervical cancer prevention in African American women. The second objective was to determine the suitability and effectiveness of the new curriculum among participants attending a NWP educational programme at three pilot sites prior to its dissemination and implementation throughout the NWP network. A unique aspect of this study is the capacity building that occurs at both the community partner level (ie NWP sites) and at the community participant level (ie African American women). Both levels of engagement are central to a community‐based participatory research (CBPR) approach and in line with the mission of the original NWP model. These objectives were accomplished using a progressive, four‐phased approach. What follows is a report of the results and findings from each of the four phases.

## 2 METHODS

Phase I focused on revising the curriculum using a CBPR approach that involved multiple and iterative processes in which the community partners worked alongside the scientists to review the curriculum. The next phases related to a pilot implementation study of the new curriculum at three NWP sites and dissemination of the pilot study results to the national members. More specifically, phase II involved training NWP staff at three sites to deliver the new curriculum as part of the CBPR education programme, and phase III examined its acceptability, relatability and ability to educate participants attending a NWP event. Phase IV involved dissemination of the new education curriculum to NWP members attending the Annual Meeting for Education and Networking (AMEN) and subsequently revising the curriculum in light of their evaluation and feedback.

### 2.1 Sites and communities for testing the NWP curriculum

The pilot study of the feasibility and educational effectiveness of the new curriculum was conducted across three established NWP sites located in Buffalo, New York (WNY), New York City (NYC) and Little Rock, Arkansas (AR). These sites were chosen because of established research collaborations with investigators and their ability to conduct the pilot implementations of the new curriculum in less than three months. Also, training requirements were minimal in WNY and NYC as both were versed in the technology used during data collection.

The population of African American individuals in each of the study locales is comparatively higher than the national average of 12.2%.^20^ In Buffalo (Erie County), 37.3% of the population is African American (~97 000 individuals).^21^ Breast cancer mortality rates in this minority population are higher than other minorities and also the highest in New York State.^22^, ^23^ The more than two million African Americans living in NYC represent 24.4% of its population.^22^ Breast cancer mortality rates are higher in African American than white women in this city, although its breast cancer disparities mirror national trends.^23^, ^24^ The AR site covers the eastern Mississippi River Delta region and currently serves 14 predominantly rural counties comprised of mostly very poor African American individuals.^25^, ^26^ When compared to white women in the Delta region, or even other African American women from other areas within the Delta region, a similarly disadvantaged region (ie Appalachia), and the national average, the highest breast cancer mortality rates are in the African American women in AR’s service area.^25^

### 2.2 Study implementation

**Figure 1:**
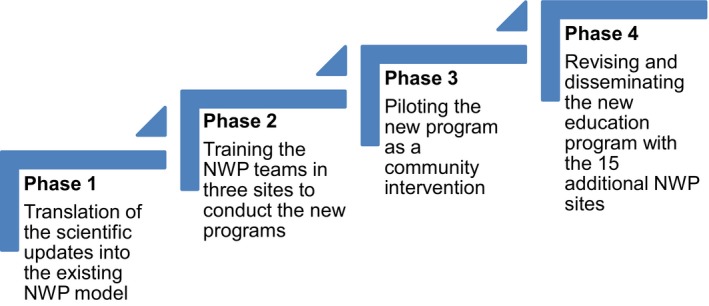
Witness project pilot study phases

Figure 1 depicts the four phases of the study design. All NWP sites are directly connected to the NWP curriculum and model and maintain the community‐based LHA‐led educational programme format including Witness Role Models (WRM).^27^ Within the context of the NWP education programme model, the LHA is a peer educator that discusses the importance of breast health awareness and also provides information about prevention, early detection and breast and cervical cancer screening services. Additionally, local African American breast and cervical cancer survivors who serve as a WRM present their personal cancer experience as a component of the educational programme. The survivor (WRM) narrative often incorporates a spiritual context and is focused on the need for early detection and treatment.

The research was conducted by local staff and volunteers at the three site locations with support from the cancer centre. Study procedures were approved by the research ethics board (WNY) and deemed as exempt by IRB (NYC). The AR site is a community‐based organization without affiliations to an academic or cancer centre and was included in the Institutional Review Board approval.

### 2.3 Phase 1: updating the national witness project curriculum

In this phase a one‐day collaborative forum was held in May 2016. Investigators, together with four scientific consultants and NWP site staff, met to update the original NWP education curriculum, its presentation to the community, and develop training material for the LHAs. The consultants were selected for their expertise in breast cancer epidemiology, African American cancer disparities, social and behavioural science, vaccine‐related cancer prevention (eg HPV) and cancer genetics. Prior to this undertaking, all had previously contributed to the NWP evolution, dissemination and implementation efforts.

Based on contributions from the NWP Board of Directors and staff members, the original narrative communication^28^ methods (eg role model stories of breast cancer experiences and early detection) used in the NWP model remained the same in the revised education programme, although the video segments were updated. The following evidence‐based changes were added to the facts presented during the breast and cervical cancer risk and screening education portion of the intervention: (a) optional use of MRI for the diagnosis of TNBC in high‐risk African American women^29^; (b) AMBER Project results showing relationship between TNBC, parity and lactation in African American women^11^, ^14^; (c) the effectiveness of the HPV vaccine in cervical, penial, anal, and head and neck cancer prevention with an emphasis on increasing vaccination rates; and (d) current breast and cervical cancer screening guidelines (United States Preventive Services Rask Force) and nature of any changes.^30^ The revisions were made using an iterative and collaborative approach guided by CBPR methods. Lastly, feedback from the NWP Board of Directors, staff and volunteers on the final products was elicited and revised accordingly.

### 2.4 Phase 2: training the trainers

Training for volunteers to deliver the new education programme was organized and conducted locally at each site by NWP leadership (Erwin and Johnson) over a 2‐month period. A minimum of five LHA volunteers at each site (WNY, NYC and AR) attended training. This study used the Turning Point Audience Response System (ARS) (Turning Technologies LLC, Youngstown, OH) polling software which allowed the LHA to ask interactive questions, track participant progress and receive instant feedback. Participants used a clicker to answer questions that were read aloud by the LHA during the education programme. The AR site had not previously used laptops, PowerPoints or embedded videos to deliver their community‐based education programme, nor were they familiar with data collection using the polling software. Thus, AR received training in the use of these technologies to increase their capacity. Additional training time and booster sessions were required to train LHAs at the AR site on using the ARS format to deliver the education programme presentation; however, the uptake of the ARS technology expedited data collection during programme delivery and data transfer for analysis following the presentations. Specific to the delivery of the new curriculum, this training involved didactic presentations, open discussions and role play/programme practices. In order to evaluate trainees’ understanding of the new content and their ability to answer any questions that may arise as they deliver the community education programme, trainees were given a knowledge survey (n = 9 items) about the curriculum before and after training. The polling software shows response data for each item, thereby allowing trainers to spend more time on content that requires further discussion to clarify and ensure understanding of the material to determine readiness of the LHA to deliver the education programme. The survey included items on screening guidelines and modifiable (ie breast‐feeding, HPV vaccination) and non‐modifiable (ie genetic) breast and cervical cancer risk factors. For example, true/false questions such as ‘African American and white women get breast cancer at about the same rate’ and ‘HPV is transmitted only through sexual intercourse’ were used. Information about the trainees’ role was also collected and included items to determine how long they have been with working with the NWP team, as well as their familiarity with and capacity to use technology. Lastly, trainees were also certified in research ethics through the Collaborative Institutional Training Initiative (CITI) programme 2016^31^ and familiarized with the process of informed consent. Investigators ensured the readiness of each site to execute quality CBPR.

### 2.5 Phase 3: pilot implementation of the new curriculum

For the CBPR pilot implementation, each site delivered a minimum of nine community educational programmes between October 2016 and January 2017. Programmes were held in churches, community centres or other accommodating facilities and were open to all adults wanting to attend. Inclusion criteria for the pilot study (ie completion of the pre/post programme assessments) limited participants to African American women ages 18 and above. Staff read a study information sheet aloud to eligible participants prior to initiating the education programme and allowed time for any questions about the study. Those who agreed to participate provided verbal consent and were given a copy of the study information sheet.

A contact card was distributed to participants to collect their demographic information, recent breast and cervical cancer screening history (ie ≤12 months and ≤3 years) and willingness to be contacted in the future for other programmes and/or research. Subsequently, pre‐ and post‐programme questionnaires were used to assess their breast and cervical cancer‐related knowledge, perceived risk and screening‐related behavioural intentions (ie attitude, self‐efficacy, intent). Item measures were presented as questions or statements on PowerPoint slides and were also read aloud to address any literacy issues. Sample items included ‘The risks and causes of breast cancer are the same for African American and white women’ and ‘HPV causes cervical cancer’ with ‘True/False/Don't Know’ as response options to choose from. Self‐efficacy items were ‘I can make an appointment for a …mammogram …Pap test’ and ‘I know how to go about getting a …mammogram …Pap test’ with response options on a five‐point Likert scale ‘1—Strongly disagree’ to ‘5—Strongly agree’. Screening behaviour‐related intent items included ‘Do you plan to have a …mammogram …Pap test in the next 12 months?’ (Yes/No/Not sure/Not applicable); and ‘How likely are you to get a …mammogram …Pap test in the next 12 months?’ with response options on a scale ‘1—Not at all likely’ to ‘6—Not applicable’. The post‐programme survey included questions from the pre‐survey in addition to items about the participant's satisfaction and impact of the educational content, for example: ‘Much of the information in this programme was new to me’ with response options on a scale ‘1—Strongly disagree’ to ‘5—Strongly agree’. An item to assess intent to screen as a result of programme attendance was also included: ‘Attending this programme has influenced my thoughts on getting screened’ with response options on a scale ‘1—Very much’ to ‘5—Not at all’. Responses were collected electronically using ARS keypads and then linked to their corresponding contact card. Summary scores were calculated from participant responses to 11 multiple choice knowledge items including six breast cancer and five cervical cancer item. Paper versions of the questionnaire were available if needed.^32^

Participants who were eligible for breast and cervical cancer screening were contacted at 2 weeks following the educational programme to assess knowledge, retention, dissemination, risk perception, self‐efficacy and intent for breast and cervical screening. Participants who were non‐adherent to screening guidelines were contacted again at 2 months to assess dissemination of programme content and screening intent/completion.

### 2.6 Phase 4: dissemination of the new curriculum

All existing NWP sites (n = 18) were invited to send their Project Directors and up to five LHAs and/or Witness Role Models to the NWP Annual Meeting for Education and Networking (AMEN) to be introduced to the new curriculum, shown the results of the pilot implementation CBPR, and contribute to dissemination plans. AMEN was a three‐day conference hosted by the WNY site. The first day included a two‐hour plenary session where the science and research behind the changes to the education curriculum changes were presented by the investigators and consultants. All presentations were tailored to the lay audience. Day two began with presentations of the new curriculum followed by results from the pilot implementation study. At the end of this session, an evaluation survey was distributed and conference attendees were asked to rate the new curriculum, content and format on various aspects such as presentation configuration, use of technology and length. Ratings were collected on a five‐point Likert scale ranging from ‘1—Not at all acceptable’ to ‘5—Completely acceptable’. Attendees were also asked about their beliefs about the importance of the new curriculum's content. After posing the general question ‘How important are the new messages on breast and cervical health that are included in the program to your health and/or the future health of your family?’, a total of 13 messages that collectively summarized the revised curriculum content were shown separately. Attendees were asked to rate each message on a four‐point Likert scale ranging from ‘1—Not important’ to ‘4—Essential’. Basic demographic information, age (18‐39; 40‐49; 50‐64 years of age) and educational attainment (Grade 12 or GED/high school graduate; some college or technical school; College graduate; Post‐graduate) were collected.

The remaining AMEN sessions included various organizational workshops to support the NWP sites on strategies to improve communication and collaboration, grant writing tips, board development activities and volunteer support. A breakout session exercise was also conducted using a small group discussion format led by a facilitator to generate site‐specific strategic implementation plans.

### 2.7 New curriculum: product of phases 1‐4

The updated curriculum includes a set of PowerPoint presentation slides with embedded videos for LHA‐led educational instruction with community participants. The final education programme was shown from laptops by the trained LHAs. The programme included ‘talking points’ for each slide to support the LHA in their presentation. Pre/post knowledge and screening assessments were included to reflect the changes made to the educational programme. New content on risk and opportunities for prevention regarding breast and cervical cancer relevant to African American women were presented in lay terms. A graphic representation of an African American medical doctor was also inserted throughout the presentation to emphasize and reinforce the importance of consulting one's health‐care provider to further discuss and understand their own personal risk. The two short (7.5 minutes each) educational videos were embedded into the programme presentation as part of the newly revised electronic media format. The first video covered the history and background of the NWP education programme and overview of the African American woman's experience with breast and cervical cancer, and the second focused on research findings from the AMBER study specific to the role of breast‐feeding in reducing breast cancer risk among African American women, including scientists (C. Ambrosone and J. Palmer) speaking about the results of their research.

### 2.8 Data analyses

Study site baseline characteristics were compared across all three study sites (WNY, AR and NYC). Chi‐square was used for categorical values, and one‐way ANOVA was used for all continuous values. Scores from pre/post knowledge tests were computed as the average per cent correct and the mean per cent change scores of responses to each type of question (breast and cervical) and across existing vs new educational content. Comparisons of pre‐/post‐test scores were made using paired sample *t* tests for both the training and education programme surveys. Responses from 29 participants from phase 3 (20 participants from Buffalo; nine participants from NYC) were excluded from the final analyses as they did not meet eligibility criteria for race/ethnicity and gender. The final analysis sample had a total of 303 participants including 139 from WNY, 104 from AR and 60 from NYC. Responses from the AMEN survey were summarized using descriptive statistics. All analyses were conducted using SPSS 21.0 software (IBM SPSS Statistics for Windows, version 21.0. IBM Corp).

## 3 RESULTS AND FINDINGS

### 3.1 Pre/post train the trainer survey results (Phase II)

Training across the three pilot sites resulted in a total of 24 trained individuals. There were seven LHAs, four role models, seven staff and six additional volunteers. The overall mean for per cent correct pre‐ and post‐training scores were 47.7% (SD: 21.87) and 79.2% (SD: 16.14). Pre‐/Post‐test scores indicate that participants scored significantly higher on the post‐test with a mean per cent change score of 31.5% (*P* < .000). Scores by site are presented in Table 1.

**Table 1:** Pre‐post scores witness project volunteer training (study phase 2)

	Western New York (n = 10)		New York City (n = 10)		Arkansas (n = 4)		Total (n = 24)	
	M	SD	M	SD	M	SD	M	SD
Percentage correct pre score	56.68	23.70	42.20	20.84	38.87	14.36	47.68	21.87
Percentage correct post score	77.79	18.14	84.46	14.98	69.47	10.62	79.18	16.14

Note

Paired samples *t* test was used to compare pre‐/post‐test scores, *P *= .000.

### 3.2 Pre/post pilot implementation survey results (Phase III)

A total of 31 educational programmes were piloted (WNY = 13, AR = 9 and NYC = 9) and reached a total of 332 African American female participants. Comparisons of baseline characteristics across the three study sites and overall are presented in Table 2. The average number of participants attending the programmes was 12.6 (SD: 5.18) participants, with significantly higher number of participants per programme in WNY as compared with the other two study sites (14.4 vs 13.5 AR and 7.8 in NYC; *P* ≤ .001). The majority of participants were over the age of 50 (56%) across all three sites. Almost 60% of programmes were conducted during the week, and almost half of the programmes were in the afternoon and lasted on average 80 minutes.

**Table 2:** Witness project pilot programme summary by national site (study phase 3)

Variables	New York City (n = 60)	Western New York (n = 139)	Arkansas (n = 104)	Total (n = 303)	*P‐value*
	n (SD)	n (SD)	n (SD)	n (SD)	
Total number of programmes	9	13	9	31	
Total participants (mean)	7.8 (3.95)	14.4 (5.38)	13.5 (3.64)	12.6 (5.18)	.000*
Age range					
18‐39	10	24	30	64	.073**
40‐49	11	16	26	53	
50+	32	71	47	150	
Day of the week					
Weekday	3	10	5	18	.124**
Weekend	6	3	4	13	
Programme time					
Morning	1	5	1	7	.364*
Afternoon	5	6	3	14	
Evening	1	2	4	7	
Programme Length (mins)	76.7 (20.46)	79.6 (9.46)	83.9 (6.01)	80.1 (12.85)	.169*
Number of Volunteers (mean)	1.2 (1.34)	2.5 (1.27)	3.6 (23.77)	12.7 (5.18)	.001*

* One‐way ANOVA was used to analyse continuous variable.

** Chi‐square test was used to test categorical variables.

The overall mean and mean per cent change scores for pre/post knowledge breast and cervical cancer items including original and new educational content are presented in Table 3. Overall change in knowledge scores (both breast and cervical cancer items) indicates that participants scored significantly higher on the post‐test (84.4 Mean % vs 55.3 Mean %) with a mean per cent change score of 29% (*P* ≤ .001). For breast cancer items, participants also scored significantly higher on the post‐test (88.7 Mean % vs 57.7 Mean %) with a mean per cent change score of 35.9% (*P* ≤ .001). Similarly, for cervical cancer items, participants knowledge increased on the post‐test, with an average mean per cent correct score of 94.4% with a mean per cent change score of 36.8% (*P* ≤ .001). For new items covering content specific to breast‐feeding, parity and HPV virus, participants displayed an increased score in the post‐test (92.4 Mean % vs 43.7 Mean %), with a mean per cent change score of 48.7% (*P* ≤ .001).

**Table 3:** Pre‐post Scores for Original and Revised Educational Content (Study Phase 3)

	n	Mean % (SD)	Mean % Change Score (SD)	*P*‐value
Total pre scores (11 items)	140	55.3 (19.8)	29.0 (18.7)	.000
Total post scores	140	84.4 (10.1)		
Breast items pre score (6 items)	164	52.8 (21.7)	35.9 (1.8)	.000
Breast items post score	164	88.7 (15.4)		
Cervical items pre score (5 items)	180	57.7 (36.3)	36.8 (1.9)	.000
Cervical items post score	180	94.4 (13.2)		
Original items pre score (6 items)	149	65.9 (26.8)	26.1 (2.0)	.000
Original items post score	149	92.0 (12.3)		
New items pre score (5 items)	174	43.7 (24.7)	48.7 (26.3)	*.000*
New items post score	174	92.4 (15.6)		

* Paired samples *t* test was used to calculate mean per cent differences and significance.

Although the primary aim for the pilot was feasibility and increase of knowledge by participants, telephone follow‐up surveys with a limited sample (20%) of previously non‐adherent women attending the new education programmes demonstrated a 33% screening rate two months later.

### 3.3 Annual meeting for education and networking evaluation survey results (Phase IV)

Evaluation survey results regarding the new curriculum content were collected from participants in attendance during the AMEN and are summarized in Table 4. A total of 103 participants from 14 NWP sites attended the AMEN. As shown, the average mean range for the 10 items regarding acceptability of curriculum content and format ranged from 4.23 to 4.65 and the average was 4.61 out of 5. The average mean for the breast cancer items was 3.82 out of 5, and the average mean for cervical cancer items was 3.86 out of 5. The two lowest rated items on new breast cancer information had average means of 3.72 and 3.74, respectively, and covered information on incidence and parity specific to African American women.

**Table 4:** Results from AMEN survey for NWP Site Participants (n = 103) (Study Phase 4)

		n	%
Age range (in years)	18‐39	10	10
	40‐49	6	6
	50‐64	36	35
	Missing	51	49
What is the highest grade or year of school you completed?	Grade 12 or GED (high school graduate)	3	3
	Some college or technical school	15	15
	College graduate	23	22
	Post‐graduate	18	17
	Missing	44	43
		**Mean**	**Range**
How would you rate the new program content and format?*10 items*	Powerpoint Presentation Format	4.59	3‐5
	Use of Audience Response System (ARS) Technology	4.52	3‐5
	Videos included in the presentation	4.50	3‐5
	Use of the African American female doctor to reinforce information on the slides	4.46	2‐5
	Picture and graphics used throughout the presentation	4.52	3‐5
	Length of the presentation	4.23	1‐5
	Questions asked in the pre‐ and post‐test evaluation	4.57	2‐5
	Breast cancer information overall	4.66	3‐5
	Cervical cancer information overall	4.68	3‐5
	Breast and cervical cancer information covered in the same presentation	4.65	3‐5
1—not at all acceptable; 2—slightly acceptable; 3—moderately acceptable; 4—very acceptable; 5—completely acceptable.			
		**Mean**	**Range**
How important are the new messages on breast and cervical health that are included in the program to your health and/or the future health of your family? *13 items*	African American women get breast cancer as often as white women	3.72	2‐4
	African American women are more likely to get breast cancer at an earlier age	3.80	3‐4
	African American women are more likely to have a more aggressive type of breast cancer and die of the disease	3.88	3‐4
	Having multiple children (parity) increases breast cancer risk for African American women	3.74	1‐4
	Breast‐feeding helps lower the risk of breast cancer	3.85	3‐4
	We need to encourage our young African American women to breast‐feed	3.87	3‐4
	HPV is the most common sexually transmitted infection	3.80	3‐4
	13 types of HPV are known to cause cervical cancer	3.76	2‐4
	There are 3 vaccines that can prevent the strains of the virus that cause cervical cancer	3.85	3‐4
	HPV can be transmitted by any skin‐to‐skin sexual contact	3.86	3‐4
	HPV vaccine is SAFE	3.87	2‐4
	HPV vaccine is recommended for both boys and girls	3.87	2‐4
	It's important to be proactive and get the HPV vaccine	3.85	2‐4
1—not important; 2—somewhat important; 3—very important; 4—essential.			

## 4 DISCUSSION

We conducted a CBPR approach including four phases to update the NWP educational curriculum and evaluate its effectiveness among participants who attended education programmes as part of a pilot study. The first phase resulted in the production of the updated NWP curriculum. The curriculum includes a set of PowerPoint presentation slides with embedded videos for LHA‐led educational instruction with community participants as well as updated training materials for train the trainer instruction with LHA and WRM volunteers. The second phase involved training the trainers on the new curriculum at each of the three participating pilot sites. A total of 24 individuals were trained across the three pilot sites. Trainees scored significantly higher on the post‐test with a mean per cent change score of 31.5% (*P* < .000).

In the third phase, the new education curriculum was implemented across three NWP sites as a pilot study to evaluate effectiveness of the curriculum among community participants. A total of 31 educational programmes were conducted across the three NWP sites reaching a total of 332 African American female participants. The overall mean and mean per cent change scores for pre‐/post‐test breast and cervical cancer knowledge items including newly added content all increased significantly on the post‐test in comparison with pre‐test scores. During the fourth phase, the new curriculum was disseminated at the AMEN. A total of 103 participants attended the AMEN from 14 of the 18 NWP sites. Evaluation results from the AMEN showed positive ratings in both the importance and acceptability of the new curriculum among the broader NWP constituency including LHA and WRM volunteers and staff.

Our findings expand upon the limited research that has previously been conducted on the delivery of faith‐based, African American‐focused LHA‐led education programmes to increase breast^33^, ^34^, ^35^ and cervical cancer health knowledge.^36^ We are the first to report on a CBPR approach to update curriculum content in order to more broadly disseminate important scientific findings relevant to breast and cervical cancer prevention and risk (ie breast‐feeding and HPV vaccination) that is also culturally tailored to African American women. Prior research on faith‐based cancer education and lifestyle interventions among racial ethnic minority groups suggest that these organizations have a unique position to deliver health information and services to underserved communities and many cancer prevention researchers have used churches as health intervention settings for cancer education especially among African Americans.^36^, ^37^, ^38^, ^39^

In addition, our findings also expand upon the NWP as a long‐standing community‐based breast and cervical cancer educational programme that targets underserved African American women nationally to increase awareness and understanding, and promote routine screening for breast and cervical cancer. As the science of cancer continues to evolve and new information is available regarding risk and behaviours to reduce risk (eg breast‐feeding and HPV vaccination), it is essential that this critical information be incorporated into this multi‐site programme in order to reach individuals in various communities at a grass roots level. This article reveals the complexity of the process necessary to update educational curriculum content for a multi‐site, community‐based outreach organization such as the NWP, as well as evidence demonstrating the need for this information in these communities. It was essential to receive perspectives, relevant content and evaluation from representatives described in each of the four phases of the research for the final product to be accurate, culturally appropriate and acceptable for dissemination.

The new NWP educational curriculum was shown to be effective among community participants as the overall change in knowledge scores increased significantly at post‐test. Scores increased consistently across both the breast and cervical cancer items that were assessed. The greatest mean per cent change scores were observed for the new items that covered breast‐feeding, parity and HPV virus content. The significant pre‐ to post‐education knowledge scores (*P*‐values all ≤0.001, mean change 10%‐36%) clearly demonstrated that this was new information to participants at these sites—both community members and NWP volunteers/staff. This scientific content provides new opportunities to increase awareness and understanding regarding breast and cervical cancer risk relevant to African American women. The information on the protective benefits of both breast‐feeding and the HPV vaccination relates to behaviours that either occur or are recommended at younger ages in comparison with relevant cancer screening ages for both breast and cervical cancer and therefore apply to a broader age group of women. The new educational content on breast‐feeding and HPV vaccination serves as an opportunity to expand the focus for conducting the NWP programme and outreach with multi‐generational African American audiences.

To our knowledge, this is the only study that has tested a community‐based educational curriculum using the NWP model to deliver new scientific content (eg breast‐feeding, parity and the HPV virus) regarding breast and cervical cancer risk and opportunities for risk reduction targeting underserved African American women. Many of the women in these communities were unaware of or very few had been exposed to information on the cancer prevention benefits of either of these primary prevention strategies prior to our pilot study. Despite the important strides that continue to be made in the science of cancer, there are challenges in how to disseminate new information on important advances to achieve health equity at the community level. Part of the challenge in disseminating the new NWP content relates to its acceptability among women in the African American community as was demonstrated in the AMEN evaluation survey results. The information on parity (ie higher parity without breast‐feeding associated with higher risk of TNBC) showed the most variability with respect to importance. This highlights the importance of credible messengers and decades of experience the NWP has amassed through their community‐based LHA‐led cancer prevention efforts targeting underserved African American women.

## 5 LIMITATIONS

There are limitations to our study as this was not a randomized control trial at the site or programme level. In phase 2, differences in the trainees’ pre/post knowledge assessment scores across the sites may have been due to the different sizes of training classes at each site. During phase 3, the pilot implementation of the new curriculum, pre/post programme assessments helped to measure changes in knowledge but are not an indicator of participants’ ability to retain the information or their actual behaviours with regard to breast and cervical cancer risk reduction (eg breast‐feeding or HPV vaccination). We encountered missing data for either pre‐ or post‐test or for both the pre‐/post‐test for phase 3 (pilot study) as not all participants answered all questions. Therefore, we have different sample sizes for the comparisons across breast and cervical cancer as well as existing versus new content. We were limited in our collection of breast and cervical cancer screening outcomes as this was not a primary endpoint for this study. Our primary objective was to assess feasibility and test effectiveness of the new curriculum. Cancer screening outcomes were collected for a subsample of participants reached as a component of follow‐up; however, these results were not significant. The collection of participant demographic information was limited due to programme delivery‐related time constraints. Thus, we were unable to look at potential differences by level of education among participants. The addition of multiple novel topics (ie parity and breast‐feeding and HPV vaccination) required additional time to explain and allow for participant questions.

## 6 CONCLUSION

This study demonstrated that updates in scientific content and format changes can be successfully adapted into the community‐based NWP model. Our results also contribute to limited research on the critical importance of community‐based research approaches to both translate and disseminate current research findings out into the community. The NWP model remains a culturally relevant and effective way to reach African American women with breast and cervical cancer information in efforts to address existing cancer health disparities. The NWP model also demonstrates sustainability through its extensive network of sites across the United States which may serve to facilitate advocacy efforts to focus on specific cancer prevention initiatives (eg breast‐feeding) to help reduce barriers and promote the new curriculum on a national level. This network has the potential to disseminate new breast and cervical cancer prevention messages on a much broader scale and provides an opportunity to further study relevant implementation and dissemination factors in adopting the new curriculum on a larger scale.

## CONFLICT OF INTERESTS

All authors have no financial interest in this study and no conflicts of interests to disclose.

## AUTHOR CONTRIBUTIONS

Elisa M. Rodriguez; Lina Jandorf; and Deborah O. Erwin conceptualized the data. Frances G. Saad‐Harfouche and Lina Jandorf involved in data curation. Frances G. Saad‐Harfouche and Lina Jandorf formally analysed the data Deborah O. Erwin; Elisa M. Rodriguez; Detric Johnson; and Frances G. Saad‐Harfouche acquired funding. Deborah O. Erwin; Elisa M. Rodriguez; Lina Jandorf; and Detric Johnson investigated the study. Deborah O. Erwin; Elisa M. Rodriguez; and Lina Jandorf involved in methodology. Deborah O. Erwin; Elisa M. Rodriguez; Lina Jandorf; Detric Johnson; Frances G. Saad‐Harfouche; Nikia Clark; and Christy Widman involved in project administration. Deborah O. Erwin; Elisa M. Rodriguez; Lina Jandorf; Detric Johnson; Frances G. Saad‐Harfouche; Nikia Clark; and Christy Widman provided resources. Frances G. Saad‐Harfouche and Lina Jandorf assisted with software. Deborah O. Erwin; Elisa M. Rodriguez; Lina Jandorf; and Detric Johnson supervised the study. Deborah O. Erwin; Elisa M. Rodriguez; and Lina Jandorf validated the data. Deborah O. Erwin; Elisa M. Rodriguez; Lina Jandorf; Detric Johnson; Frances G. Saad‐Harfouche; Nikia Clark; and Christy Widman visualized the data. Elisa M. Rodriguez; Lina Jandorf; Frances G. Saad‐Harfouche; and Deborah O. Erwin involved in writing—original draft. Elisa M. Rodriguez; Lina Jandorf; Frances G. Saad‐Harfouche; Deborah O. Erwin; Julia A. Devonish; Detric Johnson; Anika Stewart; Nikia Clark; and Christy Widman involved in writing—review and editing.

## Data Availability

The data that support the findings of this study are available from the corresponding author upon reasonable request.
